# Identification of Milk Fat Metabolism-Related Pathways of the Bovine Mammary Gland during Mid and Late Lactation and Functional Verification of the *ACSL4* Gene

**DOI:** 10.3390/genes11111357

**Published:** 2020-11-16

**Authors:** Yongliang Fan, Ziyin Han, Xubin Lu, Huimin Zhang, Abdelaziz Adam Idriss Arbab, Juan J. Loor, Yi Yang, Zhangping Yang

**Affiliations:** 1 College of Animal Science and Technology, Yangzhou University, Yangzhou 225009, China; dx120170088@yzu.edu.cn (Y.F.); ZiyinHan@126.com (Z.H.); dx120180094@yzu.edu.cn (X.L.); hmzhang@yzu.edu.cn (H.Z.); arbabtor@yahoo.com (A.A.I.A.); 2 Joint International Research Laboratory of Agriculture & Agri-Product Safety, Ministry of Education, Yangzhou University, Yangzhou 225009, China; 3 Department of Animal Sciences, University of Illinois, Urbana, IL 61801, USA; jloor@illinois.edu; 4 Jiangsu Co-Innovation Center for the Prevention and Control of Important Animal Infectious Diseases and Zoonoses, College of Veterinary Medicine, Yangzhou University, Yangzhou 225009, China; yangyi@yzu.edu.cn

**Keywords:** Holstein dairy cow, mammary gland, transcriptome, *ACSL4*, milk fat, triglyceride

## Abstract

The concentration of bovine milk fat changes regularly with lactation stages. In particular, milk fat percentage is higher in late lactation than mid lactation. Furthermore, milk fat composition is highly subject to a few genes. Thus, transcriptome sequencing was performed to explore the expression patterns of differentially-expressed genes (DEGs) in the parenchymal mammary gland of Holstein dairy cows between mid and late lactation. The 725 DEGs were screened (fold change > 2 and *p*-value < 0.05), and the peroxisome proliferator-activated receptor (PPAR) signaling pathway associated with lipid synthesis had a significant variation between the two periods (*p*-value < 0.05). The activation of the PPAR signal pathway may a key factor in the increasing of milk fat content in late lactation compared to mid lactation. Acyl-CoA synthetase long-chain family member 4 (*ACSL4*), a member of the PPAR signaling pathway, was upregulated in late lactation compared to mid lactation (*p* < 0.05). *ACSL4* catalyzes the activation of long-chain fatty acids for cellular lipid synthesis. However, it remains uncertain that the molecular mechanism of milk fat synthesis is regulated by *ACSL4* in dairy cows. Subsequently, the function verification of *ACSL4* was performed in bovine mammary epithelial cells (BMECs). The upregulated expression of *ACSL4* was accompanied by the increase of the concentration of intracellular triglycerides, whereas knockdown of *ACSL4* decreased the concentration of intracellular triglycerides, which demonstrated that *ACSL4* plays an important role in modulating milk fat synthesis. In conclusion, the results displayed that *ACSL4* expression regulates triglyceride metabolism in ruminant mammary cells.

## 1. Introduction

Bovine milk is consumed globally by reason of its rich nutrients such as protein, fat, carbohydrate, and mineral contents [1].The milk fat concentration changes regularly with different lactation stages. In particular, milk fat percentage is higher in late lactation than in mid lactation [2]. The differential gene expression of the mammary gland occurs at different lactation stages [3,4]. Milk fat compositions are highly influenced by some genes such as large tumor suppressor kinase 1(*LATS1*) and ATP-binding cassette, subfamily A, member 1 (*ABCA1*) [5,6]. The molecular mechanism of fat metabolism is not clearly understood in the mammary gland [7]. Therefore, milk fat synthesis remains an active area of research [8]. Previous studies of milk fat synthesis have focused on gene function verification or transcriptome analysis to screen milk fat synthesis-related genes in mammary glands [3,4,9]. Few studies used a more comprehensive approach combined with transcriptome sequencing and gene function verification to enhance our understanding of underlying molecular in milk fat metabolism.

There are two main ways to synthesize milk fat: (1) the mammary gland utilizes acetic acid and β-hydroxybutyric acid as substrates to synthesize fatty acids to synthesize milk fat and (2) the mammary gland synthesizes milk fat by free long-chain fatty acids released during the degradation of triglycerides of low density lipoprotein (LDL) in the blood [10]. All fatty acids synthesize triglycerides (TG) through activation, synthesis, transport, and other enzymatic actions, subsequently, TG are released from cells to form milk fat with the help of lipoproteins [11,12,13]. About 98% of milk lipid is TG, energy-dense molecules formed by esterified three fatty acids and a glycerol backbone [14]. In milk-fat synthesis, the key step is the fatty acid activation via the catalytic action of acyl-CoA synthetase (ACS), a key milk fat synthesis enzyme [15]. Depending on fatty acid chain length and the specificity of the catalytic fatty acids, ACS is classified as acyl-CoA synthetase mid-chain (MACSL) family, acyl-CoA synthetase long-chain (ACSL) family, or acyl-CoA synthetase very long-chain (VACSL) family [16]. In recent years, the role of the ACSL family in fat synthesis has been reported gradually, but the molecular mechanism of the acyl-CoA synthetase long-chain family member 4 (*ACSL4*) regulating milk fat synthesis remains uncertain.

The aim was to analyze the transcriptome profiles in the Holstein dairy cow mammary gland during mid and late lactation and explore the effect of *ACSL4* on triglyceride accumulation in bovine mammary epithelial cells (BMECs).

## 2. Materials and Methods

### 2.1. Ethics Statement

This study was approved by the Institutional Animal Care and Use Committee (IACUC) of the Yangzhou University Animal Experiments Ethics Committee (Permit Number: SYXK (Su) IACUC 2016-0019).

### 2.2. Study Design and cDNA Library Construction and Detection

Three healthy Holstein dairy cows (A, B, and C) in their second lactation were selected from the Yangzhou University farm. Total mixed ration (TMR) was used for feeding the three dairy cows (mean weight ± SD = 628.33 ± 20.05 kg). TMR with a 55:45 concentrate-to-forage ratio contained 7% Chinese wild rye hay and 23% alfalfa hay [3]. Milk samples were collected at 180 days after calving (180 d), 210 days after calving (210 d), 240 days after calving (240 d), and 270 days after calving (270 d). Milk composition was detected using mid-infrared spectrometry (MilkoScan Minor, Foss Analytics, Hillerød, Denmark) [17]. Somatic cell counts (SCCs) in milk samples were estimated by MilkoScan (FOSS 6000, Denmark) and somatic cell scores (SCSs) was calculated according to the formula: SCS = (log_2_ SCC/10,000) + 3 [18]. The daily milk yield of the dairy cows was recorded at 180 d, 210 d, 240 d, and 270 d. Surgical methods collected dairy cow mammary glands at mid (180 d) and late (270 d) lactation [19,20]. The detailed operation was as follows. First, 35 mg SU-MIAN-XIN (846 compound anesthetic agents, intravenously) and 1 mL procaine (subcutaneously) were injected into a quarter. Then the midpoint of this quarter was cut with a 1.5 cm incision. Next, connective tissue was blunt dissected, exposing the parenchymal tissue. After that, the mammary tissues were collected and washed with diethyl pyrocarbonate (DEPC) -treated ddH_2_O. The mammary tissues were placed into a sterile tube and frozen in liquid nitrogen immediately until RNA isolation and histological observation. Paraffin sections were stained with hematoxylin and eosin (HE) for routine histological studies, and the sections were made in the same manner as Li et al. [21]. Sections were analyzed by light microscopy using a Nikon fluorescence microscope (Nikon, Tokyo, Japan).

The mirVana™ miRNA Isolation Kit (Ambion-1561) containing the DNase was used for total RNA extraction from the mammary gland. Subsequently, the quantity of the total RNA was evaluated by NanoDrop 2000 (NanoDrop, Waltham, MA, USA) [22]. In addition, RNA integrity was confirmed using the Agilent 2100 Bioanalyzer (Agilent Technologies, Santa Clara, California, USA) and 1% agarose gel electrophoresis. RNA with a ratio of 28S/18S ranging from 1.5 to 2.6 and RNA integrity number (RIN) ≥ 7 was applied for the transcriptome sequencing [3]. First, the cDNA libraries were constructed using the TruSeq Stranded mRNA LT Sample Prep Kit (Illumina, catalog #RS-122-2101, San Diego, CA, USA) and sequenced on a sequencing platform (Illumina, HiSeqTM 2500), generating raw reads with 125 bp paired-end. Then, the quality control of raw reads were performed using the NGS QC Toolkit v2.3.3 to remove unqualified reads and generate clean reads [23]. Next, sequencing saturation analysis was carried out to assess the quality of sequencing. Finally, the clean reads were mapped to reference bovine genome UMD3.1 (ftp://ftp.ncbi.nlm.nih.gov/genomes/all/GCF_000003055.6_Bos_taurus_UMD_3.1.1) using TopHat 2.1.1 (http://ccb.jhu.edu/software/tophat/index.shtml) and Bowtie 2 2.3.5.1 (http://bowtie-bio.sourceforge.net/index.shtml) [24,25].

### 2.3. Identification of Differentially-Expressed Genes

The abundance of transcripts was measured by fragment reads per kilobase per million mapped reads (FPKM) [26]. The read counts per gene were calculated on HtSeq-count 0.9.1 (https://htseq.readthedocs.io/en/master/history.html#version-0-9-1) [27]. Principal component analysis (PCA) evaluated DEG expression patterns. The differential expression analysis of transcripts from mid and late lactation was performed using the DESeq R package (1.18.0) (http://www.bioconductor.org/packages/release/bioc/html/DESeq.html) [28]. Subsequently, genes with fold change > 2 and *p*-value < 0.05 were identified as differentially-expressed genes (DEGs).

Six DEGs selected randomly were applied to quantitative real-time PCR (qRT-PCR) for the verification of transcriptome sequencing data (Table S1). All reactions had three biological replicates (a biological replicate with three technical replicate) in each test day. The reactions were performed in the Light Cycler^®^ 480 System (Roche, Indianapolis, IN, USA). A SYBR Green PCR Master Mix (TaKaRa, catalog #RR820) was utilized for detecting the fluorescent signals. All reactions had three biological sample with three replicates were performed in the Light Cycler^®^ 480 System (Roche, USA). Thermocycling conditions were as follows: 40 cycles of 95 °C for 30 s, 95 °C for 10 s, and 60 °C for 30 s. Relative gene expression was calculated using the 2^−ΔΔCt^ method [29] and normalized to the housekeeping gene RPS9 and β-actin [3,30].

### 2.4. Bioinformatic of Differentially-Expressed Genes Analyses

Hierarchical cluster analysis explored DEG expression patterns. Gene ontology (GO) annotation was executed by DAVID 6.8 [31]. Kyoto Encyclopedia of Genes and Genomes (KEGG) pathways analysis was achieved on KOBAS 3.0 [32]. R based on the hypergeometric distribution was operated to analyze not only GO terms but also KEGG pathways [33]. The calculation formula was as follows: (N represents the number of genes annotated in a pathway, n represents the number of DEGs in N, M represents the number of genes belonging to a particular pathway, and m represents the number of DEGs annotated in this particular pathway). *p*-value < 0.05 was a threshold to identify significantly-enriched GO terms and KEGG pathways. Then, a protein–protein interaction (PPI) network of DEGs was built to investigate the interactions of DEGs based on GO and KEGG enrichment analysis [34].

$$p=1-\overset{m-1}{\underset{i=0}{\sum }}\frac{\left(\begin{array}{c} M\\ i \end{array}\right)\left(\begin{array}{c} N-M\\ n-i \end{array}\right)}{\left(\begin{array}{c} N\\ n \end{array}\right)}$$

### 2.5. ACSL4 Protein Eukaryotic Expression Constructs

The two fragments (L4-1 and L4-2) were amplified by PCR using cDNA extracted as template from BMECs that were cultivated in six-well plates with the complete dulbecco’s modified eagle medium/nutrient mixture F-12 (DMEM/F-12) medium (Gibco, catalog #11330032, Thermo Fisher Scientific, Waltham, MA, USA) mixed with 10% fetal bovine serum (FBS) (Gibco, catalog #10099141) and necessary reagents (e.g., 5 μg/mL bovine insulin, 10 kU/L cyan/streptomycin) (Invitrogen, catalog #7120-30, Carlsbad, CA, USA) at 37 °C in a humid cell-culture incubator [6]. Then the two fragments were fused by overlap-extension-PCR to generate the target fragment (L4). PCR reactions were performed using the PrimeSTAR^®^ Max DNA Polymerase (TaKaRa, catalog #RR820, Beijing, China). All primer sequences used are listed in Table S2. The target fragment was inserted into pcDNA3.1(+) and digested by vector Xho I/Hind III. Gene recombination was performed using the ClonExpress^®^ II One Step Cloning Kit [35].

### 2.6. Cell Culture and Transfection

BMECs resuscitated were cultivated in six-well plates with complete DMEM/F-12 medium at 37 °C in a humid cell culture incubator. When grown to approximately 80% confluent, BMECs were transfected with the siRNA (ID: Bos-323). After 48 h, BMECs transfected with siRNA were collected for index detection. Each experiment had three biological repeats. The siRNA sequences were Bos-323-1, 5′ UCAAUAGAAUUGCCUGCU 3′ and Bos-323-2, 5′ UAAGCCCAGUGGUUUAUGC 3′.

### 2.7. Determination of Relative Gene Expression

mRNA expression was assayed by qRT-PCR according to the method mentioned in Section 2.6. Total RNA from the transfected BMECs was extracted manually using TRNzol Universal Reagent (Tiangen, catalog #DP424, Beijing, China). RNA quality was checked by a spectrophotometer (Thermo Scientific, Wilmington, DE, USA). The mRNA in qualified total RNA was reverse-transcribed into cDNA using the PrimeScript™ RT reagent kit with gDNA Eraser (Takara, catalog #RR047A, Beijing, China). The procedure was as follows: 95 °C for 30 s, followed by 40 cycles of 95 °C for 5 s and 55 °C for 30 s. Table S3 showed the primer sequences.

*ACSL4* protein expression was analyzed by Western blot. Protein extraction from the transfected BMECs were using radio immunoprecipitation assay (RIPA) lysis buffer containing phenylmethanesulfonyl fluoride (PMSF) (Solarbio, catalog #R0020, Beijing, China). Proteins were separated using sodium dodecyl sulfate–polyacrylamide gel electrophoresis (SDS-PAGE), then they were transferred to a PVDF membrane (Amersham Biosciences, catalog #IPVH00010, Amersham, Buckinghamshire, UK) and probed with the primary polyclonal goat anti-ACSL4 antibody (Abcam, catalog #ab110007) and the monoclonal mouse anti-β-actin antibody (Proteintech Group, catalog #66009-1-Ig, Manchester, UK) [36].

The expression level of lipid synthesis-related genes in BMECs by *ACSL4* knockdown and overexpression were determined by qRT-PCR. Then a PPI network was built to investigate their interactions.

### 2.8. Triglyceride Content Assay

Triglyceride content was determined by an enzymatic triglyceride assay kit (Applygen, catalog #E1013, Beijing, China). The transfected BMECs were lysed and left standing 10 min. The supernatant was collected in a 1.5 mL microfuge tube, placed in a 70 °C water bath for 10 min. Then the liquid was centrifuged at 2000 rpm for 5 min to obtain supernatant for the enzymatic assay [37,38]. Each enzymatic assay was detected with three biological replicates and each biological replicate was detected with three technical repeats. Absorbance were measured at 550 nm using a microplate reader (Tecan, Mennendorf, Switzerland, Infinite M200 Pro NanoQuant).

### 2.9. Statistical Analysis

All the statistical analyses were performed with GraphPad Prism 8. All data were shown with the mean ± standard error (SE). *p* < 0.05 (*) was considered significant. The PPI network of DEGs with combined score > 0.7 was built by STRING v11.0 and visualized by Cytoscape v3.7.2 [39].

## 3. Results

### 3.1. Gene Expression-Level Analysis

Milk composition analysis showed that the milk fat content increased from mid lactation to late lactation in the dairy cows (Table S4). The results of histological observation indicated that the samples for subsequent analysis were mammary parenchymal tissues (Figure S1). The quality parameters of total RNA extracted from the mammary tissues are shown in Table S5**.** The ratio of 28S/18S of total RNA ranged from 1.82 to 2.05 and the RINs of all RNA samples were more than 7. In addition, the electropherogram image (Figure S2) showed that the total RNA was less degraded. Then six cDNA libraries were established using the RNA extracted from the Holstein dairy cow mammary glands during mid and late lactation, generating approximately 72.61GB and 72.21GB raw reads for each library, respectively (Table S6). Raw sequence data was deposited in the Genome Sequence Archive in the BIG Data Center, Beijing Institute of Genomics (BIG), Chinese Academy of Sciences under an accession number—CRA002742 and are publicly accessible at http://bigd.big.ac.cn/gsa. After the quality control of sequencing data, the Q30 percentages exceeded 95.92% and GC content ranged from 48.00% to 50.00%. The results of the saturation for the six libraries indicated that high expression genes were easier to reach saturation than low expression genes and the current sequencing data has reached saturation for all genes (Figure S3). Among cDNA libraries, over 79.24% of the reads were uniquely mapped.

**Figure 1:**
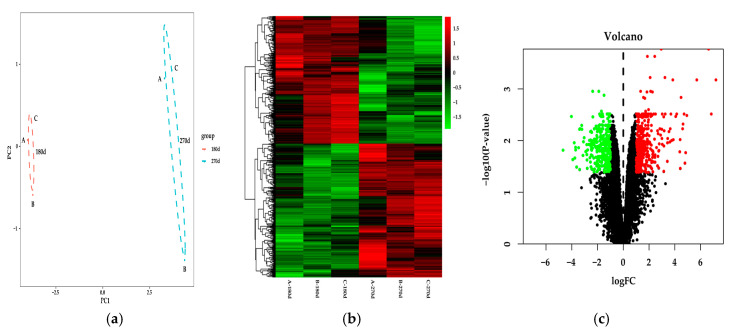
DEGs were identified in Holstein dairy cow mammary gland from mid lactation versus the cows from late lactation. (**a**) Principal component analysis (PCA) of the differentially-expressed gene (DEG) expression profiles. (**b**) Heat map of the DEGs. Green indicates downregulated DEGs and red indicates upregulated DEGs. (**c**) Volcano plot displays DEGs in Holstein dairy cow mammary glands during mid lactation and late lactation.

PCA was performed on the entire transcriptome dataset, then two clusters were generated— mid and late lactation (Figure 1a). Subsequently, a total of 725 DEGs were selected by fold change > 2 and *p*-value < 0.05. There were 371 upregulation genes, including *ACSL4*, and 354 downregulation genes in late lactation compared with mid lactation (Figure 1b,c).

**Figure 2:**
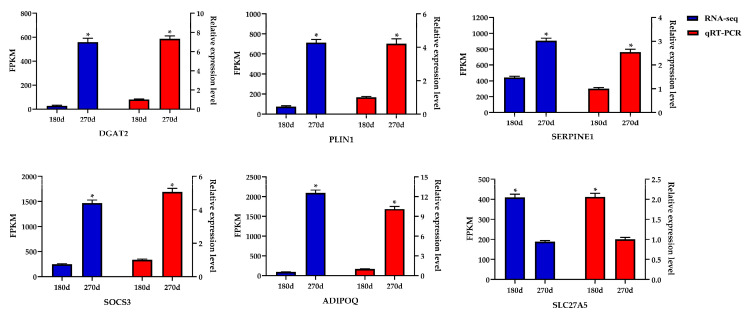
Expression level of six DEGs detected by RNA-Seq and qRT-PCR. Values are presented as the mean ± standard errors; *, *p* < 0.05.

To verify the transcriptome sequencing data, the six DEGs in the late lactation group were selected at random for qRT-PCR. The results signified that mRNA expression trends of the six DEGs were consistent between qRT-PCR results and transcriptome sequencing data. Furthermore, a highly-significant correlation coefficient (*p* < 0.001) of the two results reached 0.975 (Figure 2), indicating that the sequencing data was reliable.

### 3.2. Functional Analysis of Differentially-Expressed Genes

**Figure 3:**
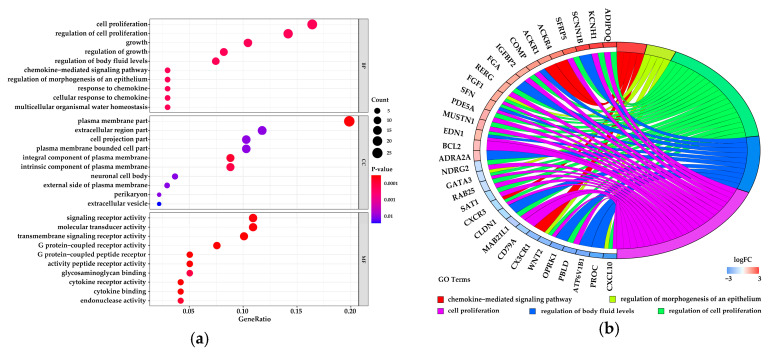

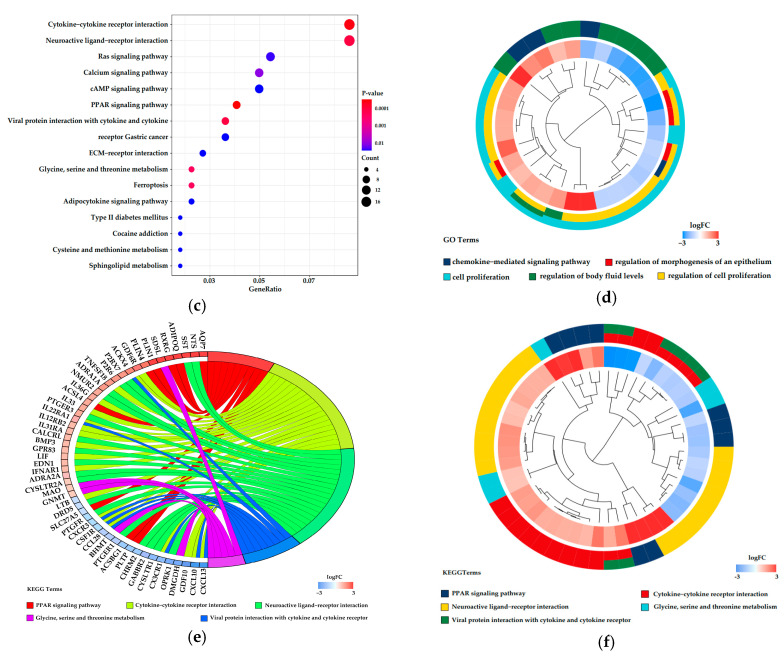
Gene ontology (GO) enrichment and Kyoto Encyclopedia of Genes and Genomes (KEGG) pathway analysis of DEGs. (**a**) Top 10 significant terms in each category were listed. (**b**) Circos plots showing overlapping and specific response of DEG-enriched top 5 significant GO terms. (**c**) Circos plot summarizing selected features of DEG-enriched in top 5 significant GO terms. (**d**) Scatter plot of significantly-enriched KEGG pathways of DEGs. (**e**) Circos plots showing overlapping and specific response of DEGs enriched in significant key KEGG pathways. (**f**) Circos plot summarizing selected features of DEGs enriched in key KEGG pathways.

The 725 DEGs were classified into three classes—biological process (BP), cellular component (CC), and molecular function (MF)—using the GO annotation. The top 10 significantly-enriched (*p*-value < 0.05) terms are shown in Figure 3a–c showed levels of variation in the DEGs of top five significant enrichment terms.

KEGG pathway analysis of the 725 DEGs revealed 16 significantly-enriched pathways (*p*-value < 0.05) (Figure 3d), in which the peroxisome proliferator-activated receptor (PPAR) signaling pathway, including *ACSL4*, had a statistical difference between mid and late lactation in the Holstein dairy cow mammary gland. The levels of variation in the DEGs belonging to significant enrichment pathways was shown in Figure 3e,f.

The PPI network was built by DEGs from the significant enrichment GO terms and KEGG pathways to investigate the interactions of DEGs (Figure S4).

### 3.3. Transfection Efficiency Analysis

**Figure 4:**
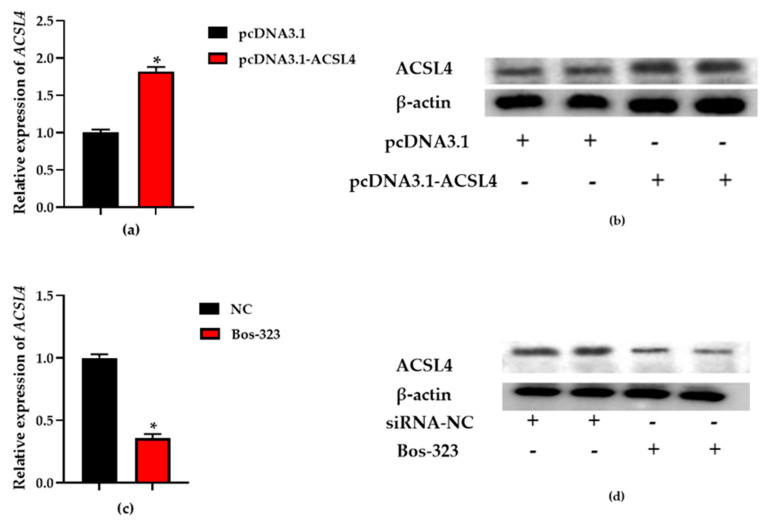
The relative expression of acyl-CoA synthetase long-chain family member 4 (*ACSL4*). (**a**,**c**) BMECs were transfected with recombinant plasmid (pcDNA3.1-ACSL4) and blank plasmid (pcDNA3.1), and the *ACSL4* expression level was quantified by qRT-PCR. (**b**,**d**) BMECs were transfected with pcDNA3.1-ACSL4 and Bos-323, respectively. The effect of pcDNA3.1-ACSL4 transfection for on *ACSL4* protein expression was evaluated by western blot. Values are presented as the mean ± standard errors; *, *p* < 0.05.

Overlapping PCR was utilized for DNA fragment (L4-1 and L4-2) joining and its products were visualized by electrophoresis through 1% agarose gels (Figure S5). To identify transfection efficiency, total RNA and protein were collected from the BMECs. Afterwards, total RNA was converted to cDNA as a template for qRT-PCR analysis. Total protein was used for *ACSL4* protein level determination by Western blot. The mRNA expression was quantified with β-actin and *RPS9* as internal controls and the protein expression was quantified with β-actin protein. qRT-PCR and Western blot results demonstrated that *ACSL4* expression levels in the BMECs transfected with pcDNA3.1-ACSL4 was significantly higher than that in the BMECs transfected with the empty vector (pcDNA3.1) (*p* < 0.05) and *ACSL4* expression levels in the BMECs transfected with siRNA was significantly lower than that in the BMECs transfected with siRNA negative control (siRNA-NC) (*p* < 0.05), which confirmed that the expression level of *ACSL4* was overexpressed and knocked down (Figure 4).

### 3.4. The Expression Level of ACSL4 Affects Triglyceride Content

**Figure 5:**
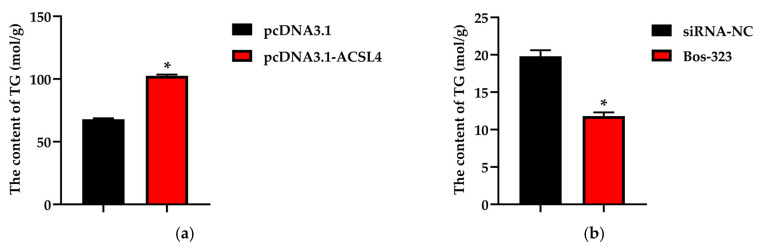
Triglycerides content assay in BMECs. (**a**) BMECs were transfected with pcDNA3.1-ACSL4, and the triglycerides content was determined. (**b**) BMECs were transfected with Bos-323, and the triglycerides content was determined. Values are presented as the mean ± standard errors; *, *p* < 0.05.

Triglyceride levels were determined in BMECs overexpressed *ACSL4*. The results demonstrated that *ACSL4* overexpression caused an increase of triglycerides content in BMECs (Figure 5a). *ACSL4* expression level was increased by 82% (*p* < 0.05) and triglyceride levels had a 51% increase in experimental group (*p* < 0.05) relative to the negative control. A siRNA for *ACSL4* was used to explore its function in BMECs. The expression level of *ACSL4* in BMECs transfected with Bos-323 decreased by 64% (Figure 5b), and triglycerides content was decreased by 25% (*p* < 0.05) compared relative to the negative control.

### 3.5. Determination of the Expression of Genes Related to Lipid Synthesis

**Figure 6:**
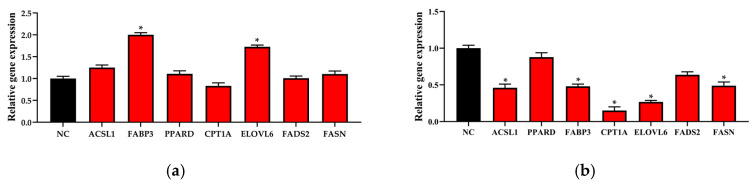
Lipid synthesis-related genes expression levels in bovine mammary epithelial cells (BMECs). (**a**) The lipid synthesis-related genes’ relative expression in BMECs after transfecting the pcDNA3.1-ACSL4 and pcDNA3.1. (**b**) The lipid synthesis-related genes’ relative expression in BMECs after transfecting the siRNA-323 and siRNA-NC. Values are presented as the mean ± standard errors; *, *p* < 0.05.

**Figure 7:**
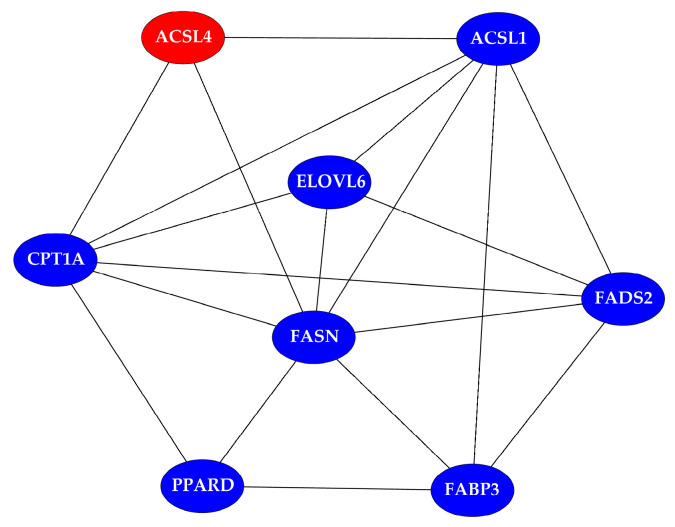
A protein–protein interaction (PPI) network of *ACSL4* protein and proteins encode by lipid metabolism-related genes whose mRNA expression was detected.

Total RNA extracted from BMECs transfected with pcDNA3.1-ACSL4/pcDNA3.1 and siRNA-323/siRNA-NC was reversely transcribed into cDNA, and the lipid synthesis-related genes expression were detected by qRT-PCR (Figure 6). The results signified that fatty acid binding protein 3 (*FABP3*) and ELOVL fatty acid elongase 6 (*ELOVL6*) expression were significantly upregulated (*p* < 0.05) and acyl-CoA synthetase long chain family member 1 (*ACSL1*), peroxisome proliferator activated receptor delta (*PPARD*), fatty acid synthase (*FASN*), fatty acid desaturase 2 (*FADS2*) and carnitine palmitoyltransferase 1A (*CPT1A*) expression were no significant differentiation in *ACSL4* overexpression BMECs (*p* > 0.05). The results signified that the mRNA expression levels of ACSL4, *FABP3*, *CPT1A*, *ELOVL6* and *FASN* were significantly downregulated (*p* < 0.05) and *PPARD* and *FADS2* expression had no significant differentiation in ACSL4 knockdown BMECs (*p* > 0.05). To investigate interactions of *ACSL4* and *ACSL1*, *PPARD*, *FABP3*, *CPT1A*, *ELOVL6*, *FADS2* and *FASN*, PPI was built by these genes (Figure 7).

## 4. Discussion

A total of 725 DEGs were identified, containing 371 upregulated genes and 354 downregulated genes in Holstein dairy cow mammary gland during late versus mid lactation. In order to obtain the potential function, GO annotation and KEGG pathways analysis was performed. KEGG analysis revealed that the PPAR signaling pathway, including 9 DEGs (*ACSL4*; phospholipid transfer protein, *PLTP*; adiponectin, *ADIPOQ*; cytochrome P450 27A1, *CYP27A1*; perilipin 1, *PLIN1*; solute carrier family 27 Member 4, *SLC27A4*; aquaporin 7, *AQP7*; acyl-Coenzyme A Oxidases 3, *ACOX3*; and perilipin 1, *PLIN4*), had significant differences between mid and late lactation (*p*-value < 0.05). In addition, previous studies have shown that milk fat content in late lactation is higher than in mid lactation [2]. Furthermore, the PPAR signaling pathway is a vital lipid metabolism pathway [40]. Therefore, the altered gene expression levels in the PPAR signaling pathway indicated that the genes might produce an effect in milk fat concentration in the mammary gland.

The ACSL family is a key enzyme family for lipid biosynthesis metabolism [41]. Many genes in the ACSL family have been found to assume regulatory roles in milk fat synthesis. Bionaz et al. [10] found that *ACSL1* expression levels continued to increase from 15 days before parturition to the 60th day after lactation in Holstein dairy cows. The study of Lin et al. [4] showed that the *ACSL1* expression level was higher in the Holstein dairy cow mammary gland during peak lactation than during the 30th day after dry off. Also, it was found that the upregulated expression level of *ACSL1* promoted triglyceride synthesis in dairy goat’s mammary epithelial cells [42]. In this study, there was no significant difference in *ACSL1* expression level between mid and late lactation and the expression level of *ACSL4*, participating in PPAR signaling pathway, was upregulated in late lactation instead of mid lactation. *ACSL4* is also a key milk fat synthesis enzyme [6,43]. However, its role is not yet clear in the Holstein dairy cow mammary gland. Thus, we explored the function of *ACSL4* in regulating the primary milk fat–triglyceride content in BMECs. We knocked down *ACSL4* with siRNA, leading to a subsequent decrease in triglyceride levels of BMECs. Meanwhile, the triglyceride level in BMECs overexpressing *ACSL4* was significantly upregulated (p < 0.05). To overexpress *ACSL4* in BMECs, we constructed a recombinant eukaryotic expression plasmid (pcDNA3.1-ACSL4) and transfected it into BMECs. As with the increase of *ASCL4* expression levels, triglyceride concentration was elevated in BMECs. In other words, the expression level of *ACSL4* could cause the change of triglyceride level in BMECs.

It was found that the upregulation or downregulation of *ACSL4* expression levels would lead to the upregulation or downregulation of *FABP3* expression levels [44,45]. FABPs regulated the fat and glucose balance by interacting with peroxisome proliferator-activated receptors (PPARs), and played an important role in intracellular fatty acid transport and metabolism [46]. A study showed that polymorphisms in *FABP4* could select for cattle producing milk with lower concentrations of saturated fatty acids and higher concentrations of unsaturated fatty acids [47]. It also has been demonstrated that *FABP3* expression was higher in mammary glands from lactating Holstein dairy cows than that from nonlactating Holstein dairy cows [2,3]. The overexpression of *ACSL4* increased the activation efficiency of fatty acids, which induced changes in the downregulated expression of *FABP3* related to fatty acid transport. *CPT1A* as a key enzyme came into play in long-chain fatty acids transport across the mitochondrial membrane for fatty acid β-oxidation [48,49]. A significant reduction in *CPT1A* expression appeared in BMECs knocked down by *ACSL4*, which might result in reduction of β-oxidation efficiency. Fatty acids are degraded to acetyl-CoA by β-oxidation [50]. *FASN*, an enzyme crucial for endogenous lipogenesis in mammals, catalyzes long-chain fatty acid formation using NADPH, malonyl-CoA, and acetyl-CoA [51,52]. A decrease in acetyl-CoA of *ACSL4* knockdown BMECs led to *FASN* expression level downregulation. However, *FASN* expression level had no significant change when *ACSL4* expression level was significant downregulated. In previous studies, the expression level of *FASN* was upregulated in the Holstein dairy cow mammary gland during lactation compared to during nonlactation [2,3]. It has been proved that the upregulated expression levels of *FASN* caused triglyceride levels to be elevated in BMECs. The expression trend of *ELOVL6* and *ACSL4* were the same during mid and late lactation. *ELOVL6* is a highly-conserved family of endoplasmic reticulum enzymes, and *ELOVL6* protein is the key enzyme to catalyze the formation of long-chain fatty acids [43]. The change of *ACSL4* expression caused a change in the content of long-chain fatty acyl coenzyme A (acyl-CoA) in BMECs. Simultaneously, this resulted in the change of *ELOVL6* protein expression which catalyzed the elongation of long-chain fatty acids [53].

## 5. Conclusions

In total, 725 DEGs were identified in Holstein dairy cow mammary gland during mid and late lactation. Functional analysis showed that the PPAR single pathway had significance in the two periods. *ACSL4* was upregulated in late lactation. The upregulation of *ACSL4* increased the concentration of triglycerides, whereas its knockdown decreased the concentration of triglycerides in BMECs.

## Supplementary Materials

The following are available online at https://www.mdpi.com/2073-4425/11/11/1357/s1. Table S1: Primers used in quantitative real-time PCR to verify transcriptome sequencing data. Table S2: Primers used in PCR for the target fragment cloning. Table S3: Primers used in quantitative real-time PCR to detect genes expression levels of bovine mammary epithelial cells. Figure S1. Histological observation of the samples for transcriptome analysis. Table S4: Milk yield, milk lactose, milk fat, milk protein, somatic cell count, and somatic cell score in different test days. Table S5: The quality parameters of total RNA. Figure S2. The electropherogram image of total RNA. Table S6: Statistics of sequencing reads and mapped reads. Figure S3. The saturation for the six libraries. Figure S4. A protein–protein interaction network of DEGs. Figure S5. Agarose gel electrophoresis of DNA fragment.
