# Supplementary material for: Identification of Milk Fat Metabolism-Related Pathways of the Bovine Mammary Gland during Mid and Late Lactation and Functional Verification of the ACSL4 Gene
Source: Genes (Basel). 2020 Nov 16;11(11):1357. doi: 10.3390/genes11111357 (PMC7696932; doi:10.3390/genes11111357)
Supplement: Supplementary file 1 [file genes-11-01357-s001.pdf]

Table S1. Primers used in quantitative real-time PCR to verify transcriptome sequencing data.

Gene	Forward Primers (5′-3′)	Reverse Primers (5′-3′)	Length (bp)	GenBank ID
DGAT2	CATGTACACATTCTGCACCGATT	TGACCTCCTGCCACCTTTCT	100	NM_205793.2
PLIN1	GTGGCACTGAGACAACATCAG	GGAGCTCTTCAAGCCTCAACT	102	NM_001083699.1
SLC27A5	TCATTCGTATCCAGGACGCC	GAACAGGGGATCAGCAACGA	107	NM_001103273.1
SOCS3	TCTGTCGGAAGACCGTCAAC	CTAAAGCGGGGCATCGTACT	104	NM_174466.2
ADIPOQ	TCCTACTTCCACCCTGACTG	GGGGGATCTTCCATCTTGTCC	132	NM_174742.2
SERPINE1	CTGCGAAATTCAGGATGCGG	GGGTGAGAAAACCACGTTGC	191	NM_174137.2
RPS9	CCTCGACCAAGAGCTGAAG	CCTCCAGACCTCACGTTTGTTC	62	NM_001101152.2
β-actin	CATCCTGACCCTCAAGTA	CTCGTTGTAGAAGGTGTG	91	NM_173979.3

Table S2. Primers used in PCR for the target fragment cloning.

Fragment	Forward Primers (5′-3′)	Reverse Primers (5′-3′)	Length (bp)
L4-1	ATGAAACTTAAGCAAAGTGTG	CAGACAGCATCATACGGACA	1324
L4-2	AATGTCCGTATGATGCTGTC	AGTTCAACAATGCGAGGCTT	919
L4	CTAGCGTTTAAACTTAAGCTTATG AAACTTAAGCAAAGTGTGCTCA	AACGGGCCCTCTAGACTCGAGTT ATTTGCCCCCATACATTCG	2136

Table S3. Primers used in quantitative real-time PCR to detect genes expression levels of bovine mammary epithelial cells.

Gene	Forward Primers (5′-3′)	Reverse Primers (5′-3′)	Length (bp)	GenBank ID
β-actin	CATCCTGACCCTCAAGTA	CTCGTTGTAGAAGGTGTG	91	NM_173979.3
ACSL4	GGCACAACAGAAAGGGGTAG	GGTTCTTCAGTTCCTTCCTT	218	XM_024988728.1
ELOVL6	GGAAGCCTTTAGTGCTCTGGTC	ATTGTATCTCCTAGTTCGGGTGC	205	NM_001102155.1
FABP3	GATGAGACCACAGCAGATG	GTCAACCATCTCCCGCACAAG	120	NM_174313.2
FADS2	CGGCAAGAAGAAGCTGAAATACCTG	TGGAAATACAAAGGGATGAGCAG	92	NM_001083444.1
FASN	GGGCTCCACCACCGTGTTCCA	GTTCTGCTGGGCCTGCAGCTG	226	NM_001012669.1
PPARγ	CGCATGAAGCTGGAATATGAG	CTTCCTCTTCTCTGCCTCTG	159	NM_181024.2
ACSL1	CTTCGCAGTGGCATCATTAG	GTCCGTAGCCTTCGTAGAAC	120	NM_001076085.1
CPT1A	CTGGACCGGGAGGAAATC	CCGAGAAGTATTAAACATGCGC	93	NM_001304989.2

Table S4. Milk yield, milk lactose, milk fat, milk protein, somatic cell count and somatic cell score in different test days (means ± SE).

Test days	180 d	210 d	240 d	270 d
Milk yield (Kg)	31.24 ± 0.06 [a]	29.43 ± 0.07 [b]	27.68 ± 0.05 [c]	26.41 ± 0.06 [d]
Milk lactose (%)	5.19 ± 0.03 [a]	4.95 ± 0.02 [b]	4.93 ± 0.02 [b]	4.88 ± 0.03 [c]
Milk fat (%)	3.52 ± 0.02 [c]	3.67 ± 0.02 [b]	3.87 ± 0.01 [a]	3.89 ± 0.02 [a]
Milk protein (%)	3.21 ± 0.02 [c]	3.28 ± 0.01 [b]	3.30 ± 0.01 [b]	3.39 ± 0.01 [a]
Somatic cell count (SCC) (10^4)	31.56	31.98	37.81	46.87
Somatic cell score (SCS)	4.66 ± 0.02 [c]	4.68 ± 0.03 [c]	4.92 ± 0.02 [b]	5.23 ± 0.03 [a]

Note: Different letters a, b, c in the same row differ significantly ($p < 0.05$) by Duncan's test.

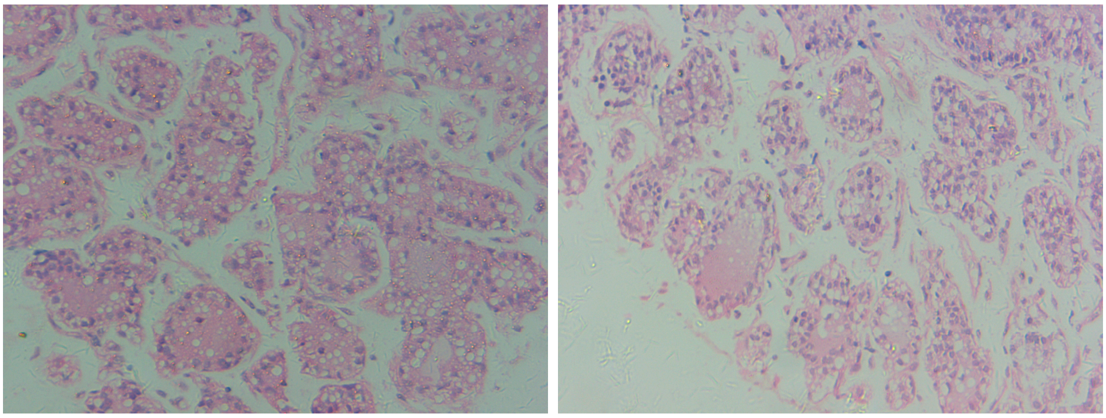

(a) (b)

Figure S1. Histological observation of the samples for transcriptome analysis. (a) (200×) Mammary gland collected during mid lactation. (b) (200×) Mammary gland collected during late lactation.

Table S5. The quality parameters of total RNA.

Sample	A260/280	28S/18S	RIN
A-180 d	2.06	2.02	7.3
B-180 d	2.05	1.82	7.5
C-180 d	2.08	1.95	7.2
A-270 d	2.07	2.01	7.4
B-270 d	2.10	2.05	7.4
C-270 d	2.10	1.87	7.2

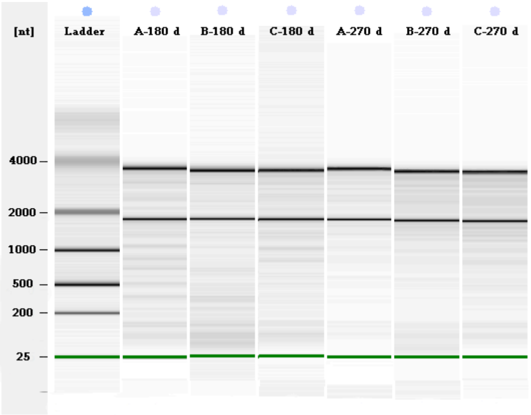

Figure S2. The electropherogram image of total RNA.

Table S6. Statistics of sequencing reads and mapped reads.

Sample	Raw Reads	Raw Bases	Clean Reads	Clean Bases	Valid Ratio (Base)	Q30	GC	Uniquely mapped
A-180 d	73996266	9.25 Gb	72947722	9.12 Gb	98.56%	96.54%	48.00%	79.24%
B-180 d	67909316	8.49 Gb	66910040	8.36 Gb	98.51%	96.33%	49.00%	84.39%
C-180 d	75935580	9.49 Gb	74705698	9.34 Gb	98.36%	96.19%	50.00%	88.85%
A-270 d	71589742	8.95 Gb	70550840	8.82 Gb	98.53%	96.48%	48.50%	81.27%
B-270 d	77932606	9.74 Gb	76857188	9.61 Gb	98.60%	96.44%	49.00%	85.30%
C-270 d	64104356	8.01 Gb	62867970	7.86 Gb	98.05%	95.92%	49.00%	88.81%

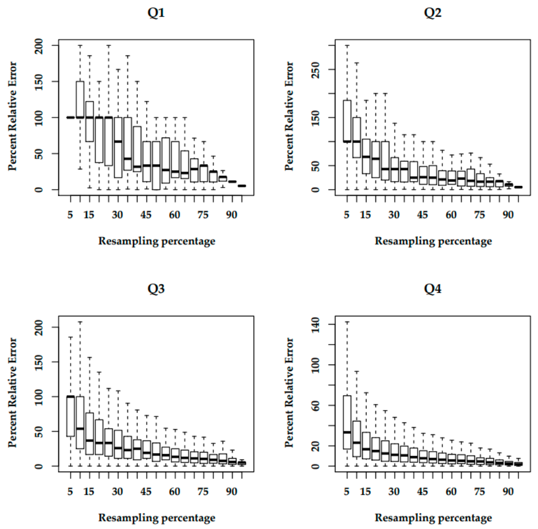

(a)

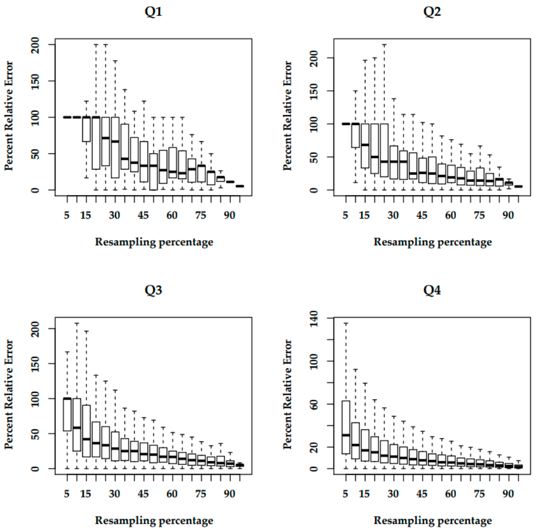

(b)

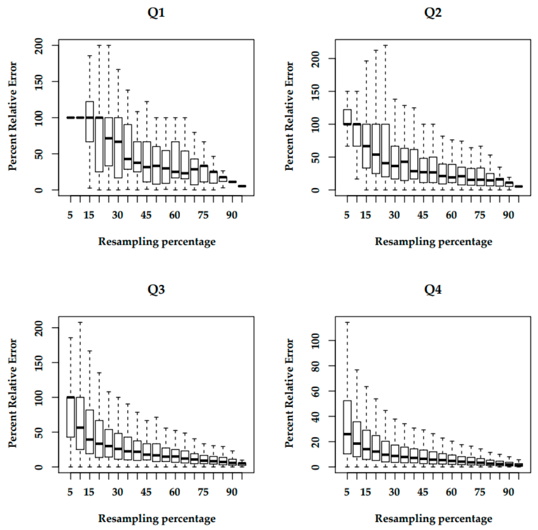

(c)

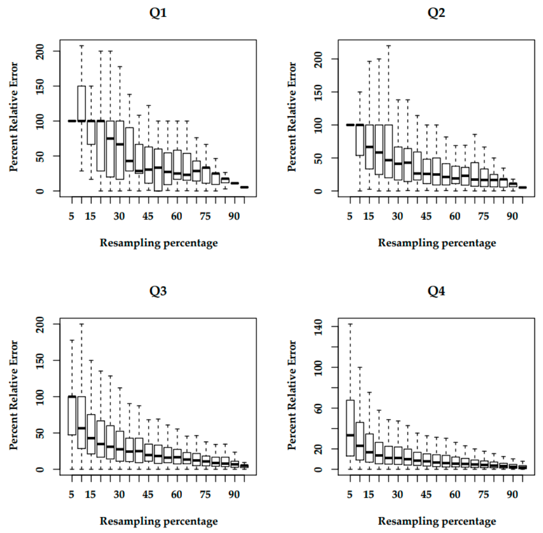

(d)

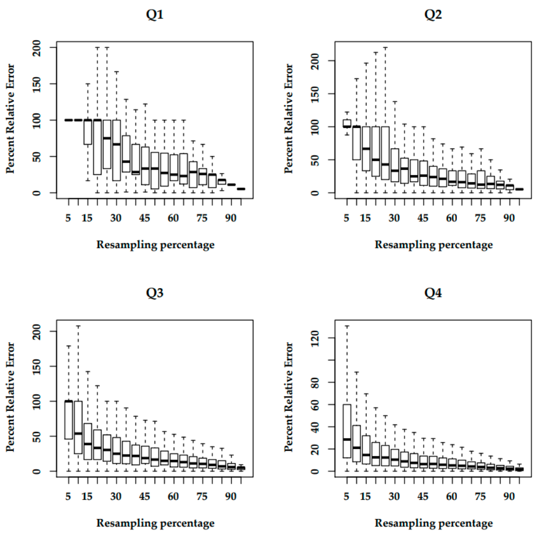

(e)

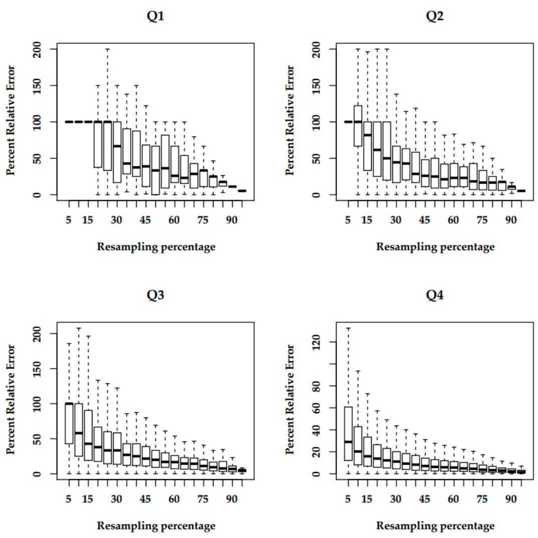

(f)

Figure S3. The saturation for the six libraries. According to their expression levels, genes (except no expression genes) were allocated in 4 groups depending on quartiles: low expression (Q1), middle expression (Q2-Q3) and high expression (Q4). (a), (b), (c), (d), (e) and (f) was for the sample A-180 d, B-180 d, C-180 d, A-270 d, B-270 d and C-270 d.

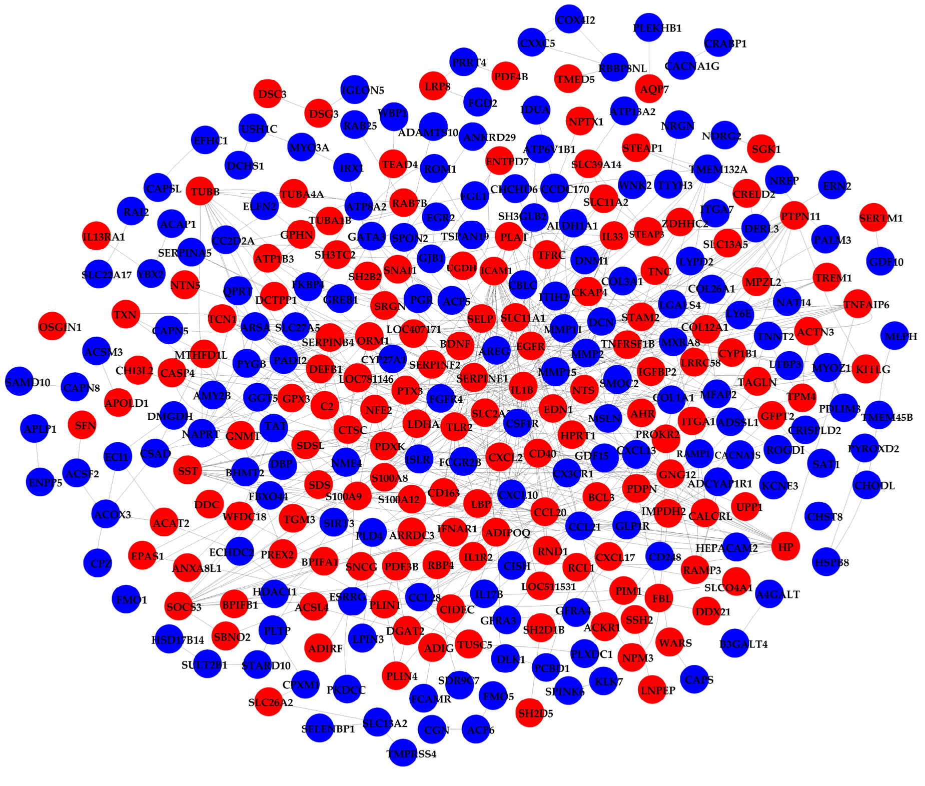

Figure S4. A protein–protein interaction network of DEGs. The node in red and blue respectively indicated that the gene was up-regulated and down-regulated in on late lactation compared with peak lactation.

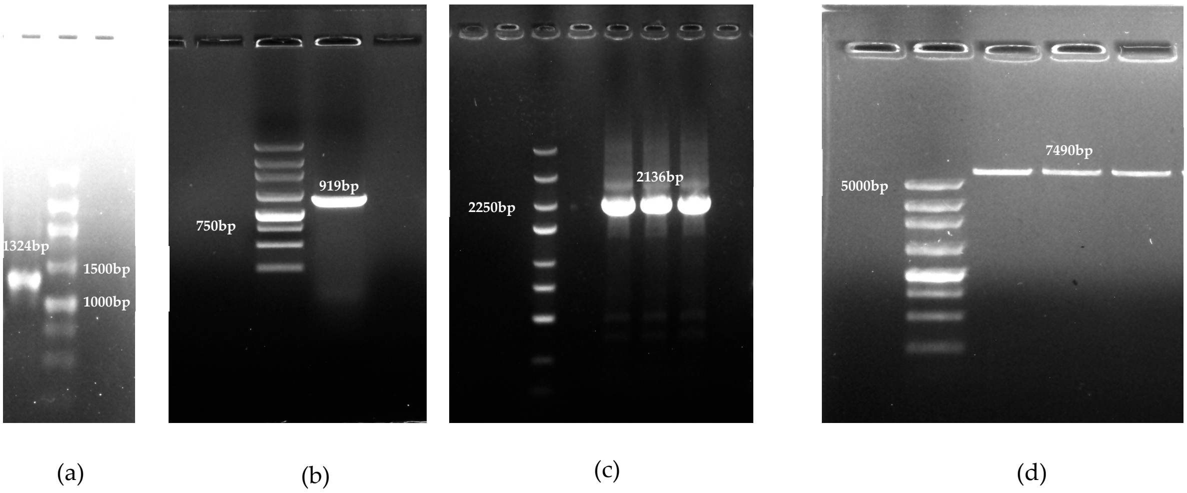

Figure S5. Agarose gel electrophoresis of DNA fragment. (a) Agarose gel electrophoresis of L4-1. (b) Agarose gel electrophoresis of L4-2. (c) Agarose gel electrophoresis of L4. (d) Agarose gel electrophoresis of linearized pcDNA3.1-ACSL4.
